# Environmental Contamination as a Risk Factor for Intra-Household *Staphylococcus aureus* Transmission

**DOI:** 10.1371/journal.pone.0049900

**Published:** 2012-11-13

**Authors:** Justin Knox, Anne-Catrin Uhlemann, Maureen Miller, Cory Hafer, Glenny Vasquez, Peter Vavagiakis, Qiuhu Shi, Franklin D. Lowy

**Affiliations:** 1 Division of Infectious Diseases, Department of Medicine, Columbia University, College of Physicians & Surgeons, New York, New York, United States of America; 2 Department of Epidemiology, Mailman School of Public Health, Columbia University, New York, New York, United States of America; 3 Panna Technologies, Inc., New York, New York, United States of America; 4 Department of Epidemiology and Community Health, School of Health Sciences and Practice, New York Medical College, New York, New York, United States of America; 5 Department of Pathology, Columbia University, College of Physicians & Surgeons, New York, New York, United States of America; National Institutes of Health, United States of America

## Abstract

**Background:**

The household is a recognized community reservoir for *Staphylococcus aureus*. This study investigated potential risk factors for intra-household *S. aureus* transmission, including the contribution of environmental contamination.

**Methods:**

We investigated intra-household *S. aureus* transmission using a sample of multiple member households from a community-based case-control study examining risk factors for CA-MRSA infection conducted in Northern Manhattan. During a home visit, index subjects completed a questionnaire. All consenting household members were swabbed, as were standardized environmental household items. Swabs were cultured for *S. aureus*. Positive isolates underwent further molecular characterization. Intra-household transmission was defined as having identical strains among two or more household members. Multiple logistic regression was used to identify independent risk factors for transmission.

**Results:**

We enrolled 291 households: 146 index cases, 145 index controls and 687 of their household contacts. The majority of indexes were Hispanic (85%), low income (74%), and female (67%), with a mean age of 31 (range 1–79). The average size of case and control households was 4 people. *S. aureus* colonized individuals in 62% of households and contaminated the environment in 54% of households. USA300 was the predominant clinical infection, colonizing and environmental strain. Eighty-one households had evidence of intra-household transmission: 55 (38%) case and 26 (18%) control households (*P<*.01). Environmental contamination with a colonizing or clinical infection strain (aOR: 5.4 [2.9–10.3] *P*<.01) and the presence of a child under 5 (aOR: 2.3 [1.2–4.5] *P* = .02) were independently associated with transmission. In separate multivariable models, environmental contamination was associated with transmission among case (aOR 3.3, p<.01) and control households (aOR 27.2, p<.01).

**Conclusions:**

Environmental contamination with a colonizing or clinical infection strain was significantly and independently associated with transmission in a large community-based sample. Environmental contamination should be considered when treating *S. aureus* infections, particularly among households with multiple infected members.

## Introduction

Numerous studies have documented the role of the household as a community reservoir for *Staphylococcus aureus* [1]–[4]. After a household member becomes infected, high levels of *S. aureus* colonization and infection often occur among other household members [5]–[8]. These reports have observed that epidemic clones tend to “ping pong” among family members, resulting in a high rate of recurrent infections [9]–[11]. Reducing the frequency of these infections in this setting has proven difficult.

Studies of household transmission of *S. aureus*, including those that focus on the spread of healthcare-associated methicillin-resistant *S. aureus* (MRSA) and, more recently, those that examined the spread of community-associated (CA) *S. aureus*, have identified a number of risk factors associated with transmission. Factors include the presence of an underlying skin condition, the sharing of items, living in a household with a previously infected member, or direct contact such as bathing an infected child [12]–[15]. In contrast with infections in the healthcare setting [16], the limited number of published studies on household transmission suggest that nasal carriage of *S. aureus* is not always associated with transmission [17].

One potential risk factor for transmission that has received limited attention in community households is the role of environmental contamination with *S. aureus*. Fomites have been identified as possible vectors of *S. aureus* transmission and infection in different settings, including the healthcare setting [18] and other community based settings, such as drug user venues [19], [20]. We recently reported that among individuals with a MRSA infection, household environmental contamination with the clinical infection strain was associated with an increased risk of antecedent infection [21]. USA300 accounted for the majority of these infections. The limited success of decolonization strategies to prevent recurrent infections in households has also raised concern regarding the role of environmental contamination in these infections [22], [23].

In light of these observations, we conducted a study to assess colonization and transmission of all *S. aureus* among the households of CA-MRSA infected case participants and healthy control participants. The study examined potential sociodemographic, health, hygiene, drug use and other household level risk factors for intra-household *S. aureus* transmission, including the contribution of environmental contamination.

## Methods

### Ethics Statement

Written informed consent was obtained from each individual before participation. Parental consent was required for the participation of children <18 years old. Pediatric assent was obtained from those capable of providing it. The Institutional Review Board of Columbia University Medical Center approved this study.

### Study Population

This analysis was part of a case-control study examining risk factors for CA-MRSA infection in the Northern Manhattan community, as defined by zip codes containing the Columbia University Medical Center catchment area. Data collection took place between January 2009 and May 2011. The overall study design has been previously described [21] and is briefly summarized below.

**Figure 1:**
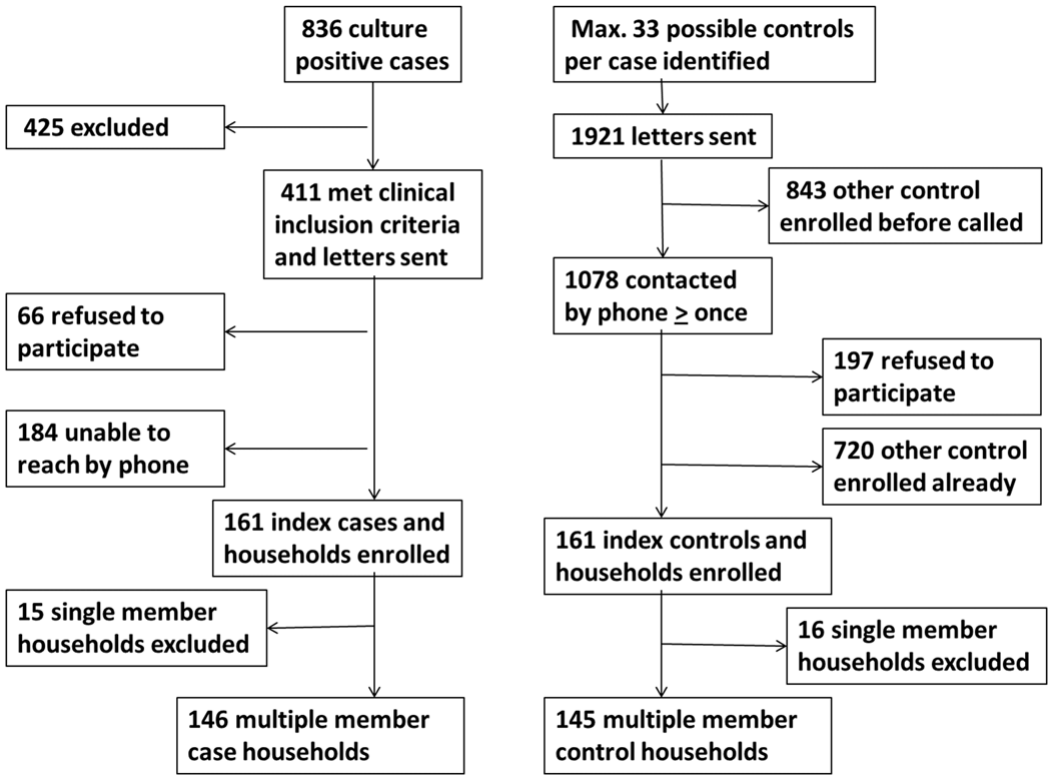
Flow chart enrollment of case and control households.

One hundred sixty one index cases with CA-MRSA infections agreed to participate in the study and were enrolled (Figure 1). Index cases were interviewed, on average, 31 days after a positive culture was obtained (sd 19; range 9–116 days). One hundred sixty one age-matched index controls identified from the Columbia University Medical Center dental clinic were enrolled in the study after undergoing the same enrollment procedures as cases. Interviews were carried out with index case and matching index controls, on average, within 28 days of each other (sd 17; range 7–90 days).

### Microbiological Specimen Collection and Molecular Analyses

The clinical infection isolates of index case subjects were retrieved from the clinical microbiology laboratory at Columbia University Medical Center. These isolates were obtained from identified sites of infection. All but one of the clinical infection isolates were located [21]. Anterior nares cultures were collected with sterile pre-moistened swabs (Becton Dickinson Culturette Systems) from all consenting household members, excluding children <1 year old (n = 52). The average number of nasal swabs collected was similar in case and control households (3.5 versus 3.2, *P* = .21).

A standardized list of environmental items were sampled with pre-moistened swabs in all households: door knobs, TV remote, living room light switch, toy, couch or bed, computer or radio, house phone or index cellular phone, bathroom sink, kitchen appliance handle. The average number of environmental items swabbed was similar in case and control households (8.7 versus 8.8, *P* = .81).

Culture swabs were incubated overnight at 37°C in high-salt 6.5% broth and plated onto Mannitol Salt Agar (Becton Dickinson) for 48 h at 35°C. Positive mannitol-fermenting yellow colonies were isolated onto 5% Sheep Blood Agar plates (Becton Dickinson) and single colonies were selected for further analysis. *S. aureus* was identified by coagulase and Protein A detection kit (Murex StaphAurex).

*S. aureus* positive isolates were genotyped by *spa*-sequencing using Ridom-staph software [24], [25]. Methicillin-resistance was assessed by presence and type of Staphylococcal Chromosomal Cassette (SCC)*mec* using multiplex PCR [26], [27]. *S. aureus* isolates characterized as *spa* type 8 with or without the presence of SCC*mec* were categorized as USA300.

### *S. aureus* Transmission Risk Factor Questionnaire

A structured questionnaire was administered to index participants to collect household-level sociodemographic information and assess risk factors for CA-MRSA, including health-related, hygiene-related, and other household-level risk factors. Potentially sensitive information was obtained using audio computer-assisted self-interviewing.

### Measures

The main outcome measure used in this study among both case and control households was *S. aureus* strain similarity within households as a proxy for transmission. Similarity was defined as having identical strains, as determined by *spa* typing, among two or more household members. Since interviews were conducted and specimens collected shortly after index cases were identified, colonization of a case household member with the clinical infection strain was additionally considered evidence of intra-household transmission, regardless of the current colonization status of the index case.

Environmental contamination with a colonizing or clinical infection strain was defined as one or more household items being contaminated with the same strain as a colonized household member or with the clinical infection strain.

### Statistical Analyses

Because the primary objective of our analysis was to assess intra-household transmission, single member households were removed from the data set. Among the 161 case households, there were 15 single member households and among the 161 control households, there were16 single member households. In order to rule out the possibility that age-matching of index cases and controls had a measureable influence on household level variables, matched pair analyses were conducted on a data set that excluded all single member households as well as their age-matched pairs. As anticipated, the results were identical; indicating that matching on the individual level had no impact on household level variables. Therefore, the final sample presented here includes 291 multiple member households: 146 case households and 145 control households.

Frequencies are presented for case index and control index participant descriptive data; chi-square tests and t-tests are used for these comparisons. In bivariate analyses comparing case and control households on sociodemographic and risk factor data, logistic regression models controlling for household size were used. In order to identify independent risk factors for intra-household *S. aureus* transmission, logistic regression models were used for both bivariate and multivariable analyses; these analyses controlled for household size and case-control status. All variables associated with intra-household *S. aureus* transmission at P<.20 in bivariate analyses were considered for inclusion in the final logistic regression model. To limit the impact of collinearity, correlations between covariates were examined. A sensitivity analysis was conducted to determine whether to include highly correlated variables. Adjusted odds ratios and 95% confidence intervals are presented. All statistical tests were 2-sided and P<.05 was considered statistically significant. Data were analyzed using SAS 9.1 software (SAS Institute Inc., North Carolina).

## Results

### Study Population Characteristics

Among the 291 index participants enrolled in the study (146 cases and 145 controls), the average age was 31 years (sd 19; range 1 to 79, *P = *.43). The majority were female (64% of cases versus 71% of controls, *P = *.23) and Hispanic (84% of cases versus 86% of controls, *P = *.65) and less than half had graduated high school (46% of case versus 38% of controls, *P = *.17).

Index participants named a total of 961 household members; of which 687 (71%) participated. The average size of case and control households was 4 people (sd 2; median 4; range 2 to 11). The mean age of household members was 27 years (sd 21; range 0 to 92 years). Fifty-one percent were female. There were no significant differences between case and control household members on any of these sociodemographic characteristics. There was no difference between case and control households in the average number of participating members (2.5 versus 2.2, *P = *.21). On the individual level, case non-index household members were more likely to provide specimens than control non-index household members [364/482 (76%) versus 323/479 (67%), (*P* = .04)].

Table 1 presents the distribution of household-level sociodemographics and risk factors among case and control households. Case households were more likely to have a higher income (*P = *.03) and share towels than control households (*P = *.05). Although there were significant differences between case and control households for variables associated with index case eligibility, control households also reported high levels of skin or soft-tissue infection (SSTI) (63%), antibiotic use (61%), and hospitalization (26%) in the past 6 months.

**Table 1:** Bivariate analyses of household-level sociodemographics and risk factors by case-control status.

	Case households		Control households				
	(N = 146)		(N = 145)				
	N	(%)	N	(%)	aOR^a^	(95% CI)	*P*
Sociodemographics							
Income < $21,000	98	(68)	114	(80)	**0.5**	**(0.3**–**0.9)**	**.02**
Child ≤5 present	64	(44)	56	(39)	1.3	(0.8–2.2)	.31
Pet present	44	(30)	42	(29)	1.1	(0.6–1.8)	.83
Travel to the Dominican Republic in the past 6 months	38	(26)	34	(23)	1.1	(0.7–2.0)	.61
Health and hygiene factors							
Surgery in the past 6 months	26	(18)	32	(22)	0.8	(0.4–1.4)	.36
Injects insulin in the past 6 months	16	(11)	7	(5)	2.4	(1.0–6.1)	.06
Home healthcare attendant	14	(10)	11	(8)	1.3	(0.6–3.0)	.54
Shares towels	38	(26)	24	(17)	**1.8**	**(1.0**–**3.2)**	**<.05**
Shares Razor	19	(13)	12	(8)	1.7	(0.8–3.6)	.19
Crowding (>1 person per room)	81	(56)	91	(63)	0.6	(0.3–1.1)	.10
Contaminated environment with a colonizing orclinical infection strain	73	(50)	43	(30)	**2.4**	**(1.5**–**3.9)**	**<.01**
Drug use and other risk factors							
Illicit drug use in the past 6 months	6	(5)	1	(1)	6.2	(0.7–52.1)	.10
HIV, IDU, MSM, Prison, STD in the past 6 months	12	(10)	7	(6)	1.7	(0.7–4.6)	.27

Abbreviations: aOR, adjusted odds ratio; CI, confidence interval; HIV, human immunodeficiency virus; IDU, intravenous drug use; MSM, men who have sex with men; STD, sexually transmitted disease.

a. Logistic regression was used to calculate adjusted OR’s and 95% CI’s, controlling for household size.

### Molecular Characterization of *S. aureus* Isolates

There was no difference in the number of individuals colonized with *S. aureus* in case versus control households [185/510 (36%) versus 157/467 (34%), *P = *.31]. Among the 185 *S. aureus* isolates colonizing case household members, we detected 64 different *spa*-types. Among the 157 *S. aureus* isolates colonizing control household members, we detected 79 different *spa*-types. There was no difference in the number of environmental items contaminated with *S. aureus* in case versus control households [198/1269 (16%) versus 164/1269 (13%), *P = *.20)]. Among the 198 *S. aureus* isolates contaminating environmental items in case households, we detected 50 different *spa*-types. Among the 164 *S. aureus* isolates contaminating environmental items in control households, we detected 47 different *spa*-types. USA300 was the most frequently cultured strain among case households. It was cultured from the nares of at least one person in 41 (28%) case households and from at least one environmental object in 36 (25%) case households. In contrast, USA300 was rarely retrieved among control households. It was cultured from the nares of at least one person in only 6 (4%) control households and from at least one environmental object in only 12 (8%) control households.

**Figure 2:**
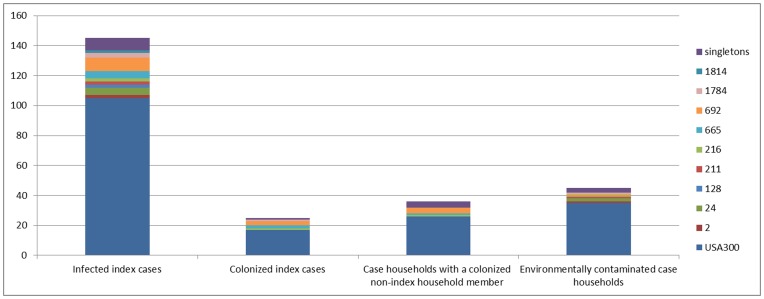
Distribution of *spa* types of 145 clinical infection isolates among case households. Distribution of *spa* types of 145 clinical infection isolates among infected index cases, colonized index cases, case households with a colonized non-index household member, and environmentally contaminated case households.

USA300 (n = 105, 72%) was also the most common clinical infection isolate. The remaining 40 (28%) clinical infection isolates belonged to 17 different *spa*-types. Less than one fifth of index cases (n = 25 or 17%) were nasally colonized with the clinical infection strain at the time of the home visit. A quarter of case households (n  = 36 or 25%) had at least one non-index member nasally colonized with the clinical infection strain. USA300 (n = 105) as compared with all other clinical infection strains (n = 40) was not significantly more likely to be found on the index (16% versus 20%, *P = *.59), a non-index household member (25% versus 25%, *P = *.95), or in the environment (33% versus 25%, *P = *.34) (Figure 2).

### *S. aureus* Colonization, Environmental Contamination, and Transmission

Including the index participants, *S. aureus* colonization was observed more frequently among the 146 case households than among the 145 control households (69% versus 55%, *P = *.02). Table 2 presents the distribution of *S. aureus* colonization among indexes, *S. aureus* colonization among non-index household members, and *S. aureus* environmental contamination by case-control status. Overall, MRSA was more likely to be collected among indexes, non-index household members and the environment in case households compared to controls. The difference in MRSA colonization was mainly accounted for by USA300. This finding was expected given the study design.

**Table 2:** *S. aureus* colonization and environmental contamination by case-control status.

	Case households		Control households				
	(N = 146)		(N = 145)				
	N	(%)	N	(%)	aOR^a^	(95% CI)	*P*
*S. aureus* colonization among indexes							
Colonized with *S. aureus*	41	(28)	50	(35)	0.7	(0.5–1.2)	.24
Colonized with MRSA	25	(17)	3	(2)	**10.3**	**(3.0**–**35.4)**	**<.01**
Colonized with USA300	18	(12)	3	(2)	**6.9**	**(2.0**–**24.3)**	**<.01**
Colonized with MSSA	16	(11)	47	(32)	**0.3**	**(0.1**–**0.5)**	**<.01**
*S. aureus* colonization among non-index household members							
Colonized with *S. aureus*	85	(58)	54	(37)	**2.6**	**(1.6**–**4.4)**	**<.01**
Colonized with MRSA	39	(27)	4	(3)	**13.4**	**(4.6**–**38.9)**	**<.01**
Colonized with USA300	28	(19)	4	(3)	**8.2**	**(2.8**–**24.2)**	**<.01**
Colonized with MSSA	60	(41)	51	(35)	1.3	(0.8–2.2)	.26
*S. aureus* environmental contamination							
Contaminated with *S. aureus*	84	(58)	74	(51)	1.3	(0.8–2.1)	.24
Contaminated with MRSA	43	(30)	6	(4)	**9.8**	**(4.0**–**24.0)**	**<.01**
Contaminated with USA300	36	(25)	12	(8)	**3.7**	**(1.8**–**7.4)**	**<.01**
Contaminated with MSSA	54	(37)	69	(48)	0.6	(0.4–1.0)	.07

Abbreviations: aOR, adjusted odds ratio; CI, confidence interval.

a. Logistic regression was used to calculate adjusted OR’s and 95% CI’s, controlling for household size.

Table 3 presents the distribution of environmental contamination with a colonizing or clinical infection strain and household transmission by case-control status. Environmental contamination with a colonizing or clinical infection strain was observed in 73 case and 43 control households (50% versus 30%, *P<*.01). Among the 73 case households with environmental contamination with a colonizing or clinical infection strain, 54 (37%) households were contaminated with the same strain as was colonizing a household member, 45 (31%) households had the clinical infection strain present in the environment, and 26 (18%) households had both the environment contaminated with the same strain as was colonizing a household member and the clinical infection strain present in the environment.

**Table 3:** Environmental contamination with a colonizing or clinical infection strain and intra-household transmission by case-control status.

	Case households		Control households				
	(N = 146)		(N = 145)				
	N	(%)	N	(%)	aOR^a^	(95% CI)	*P*
Environmental contamination with a colonizing or clinical infection strain							
Contaminated with a colonizing strain or the clinical infection strain	73	(50)	43	(30)	**2.4**	**(1.5**–**3.9)**	**<.01**
Contaminated with a colonizing strain	54	(37)	43	(30)	1.4	(0.9–2.3)	.17
Contaminated with the clinical infection strain	45	(31)					
Contaminated with a colonizing strain and the clinical infection strain	26	(18)					
Intra-household transmission							
≥2 household members colonized with identical strains or ≥1non-index household member colonized with the clinical infection strain	55	(38)	26	(18)	**3.1**	**(1.8**–**5.6)**	**<.01**
≥2 household members colonized with identical strains	35	(24)	26	(18)	1.5	(0.8–2.8)	.17
≥1 non-index household member colonized with theclinical infection strain	36	(25)					
≥2 household members colonized with identical strains and ≥1non-index household member colonized with the clinical infection strain	16	(11)					

Abbreviations: aOR, adjusted odds ratio; CI, confidence interval.

a. Logistic regression was used to calculate adjusted OR’s and 95% CI’s, controlling for household size.

Eighty-one households had evidence of *S. aureus* transmission: 55 (38%) case households and 26 (18%) control households (*P<*.01). Among the 55 case households with transmission, 35 (24%) households had multiple members colonized with the same strain, 36 (25%) households had the clinical infection strain present among a non-index household member and 16 (11%) households had both.

### Risk Factors for Household Transmission

Table 4 presents analyses assessing risk factors for *S. aureus* transmission. In bivariate models, environmental contamination with a colonizing or clinical infection strain, having a child under 5, having a pet, a household member having had surgery in the past 6 months, and crowding were positively associated with transmission at *P<*.20. Injecting insulin and travel to the Dominican Republic in the past 6 months were negatively associated with transmission at *P<*.20. In the final multivariate model, environmental contamination with a colonizing or clinical infection strain (aOR 5.4, *P<*.01) and the presence of a child less than five years old (aOR 2.3, *P = *.02) remained independently associated with intra-household transmission.

**Table 4:** Bivariate and multivariate analyses of risk factors for *S. aureus* transmission within households.

	Bivariate analyses^a,b^			Multivariate analyses^a^		
	aOR^2^	(95% CI)	*P*	aOR^3^	(95% CI)	*P*
Sociodemographics						
Income < $21,000	0.8	(0.4–1.4)	.38			
Child ≤5 present	2.4	(1.3–4.4)	.01	**2.3**	**(1.2**–**4.5)**	**.02**
Pet present	1.6	(0.9–2.9)	.13	1.8	(0.9–3.5)	.10
Travel to the Dominican Republic in the past 6 months	0.6	(0.3–1.2)	.14	0.6	(0.3–1.3)	.19
Health and hygiene risk factors						
Surgery in the past 6 months	1.9	(0.9–3.7)	.08	2.1	(1.0–4.5)	.07
Injects insulin in the past 6 months	0.3	(0.1–1.2)	.09	0.3	(0.1–1.1)	.07
Home healthcare attendant	0.8	(0.3–2.3)	.68			
Shares towels	1.4	(0.7–2.6)	.36			
Shares Razor	1.5	(0.6–3.4)	.36			
Crowding (>1 person per room)^c^	2.1	(0.9–4.5)	.07			
Contaminated environment with a colonizing or clinicalinfection strain	5.1	(2.8–9.4)	<.01	**5.4**	**(2.9**–**10.3)**	**<.01**
Drug use and other household level risk factors						
Illicit drug use in the past 6 months	2.5	(0.5–12.7)	.26			
HIV, IDU, MSM, Prison, STD	1.6	(0.5–4.8)	.41			

Abbreviations: aOR, adjusted odds ratio; CI, confidence interval; HIV, human immunodeficiency virus; IDU, intravenous drug use; MSM, men who have sex with men; STD, sexually transmitted disease.

a. Multiple logistic regression was used for analyses, controlling for household size and case-control status.

b. All variables significant at *P*<.20 in bivariate analyses were considered for inclusion in the final multiple logistic regression model to calculate adjusted OR’s and 95% CI’s.

c. Although *P*<.20, not included in the final model because of high correlation with household size.

To further explore the role of environmental contamination as a contributing factor to intra-household *S. aureus* transmission, the data were stratified by case-control status and analyzed separately. In separate multivariable models, environmental contamination with a colonizing or clinical infection strain was significantly and independently associated with *S. aureus* transmission among both case households (aOR 3.3, p<.01) and control households (aOR 27.2, p<.01).

## Discussion

This study is among the first to demonstrate that environmental contamination plays an important role in the household spread of *S. aureus*. Environmental contamination with a colonizing or clinical infection strain was significantly and independently associated with transmission in a large community-based sample.

There are now several studies that support the contribution of the environment to transmission and infection. In an earlier household-based investigation of the Northern Manhattan community, we found that environmental contamination with the clinical infection strain was associated with an increased risk of antecedent MRSA infections [21]. Additionally, a recent trial designed to reduce the incidence of recurrent CA-*S. aureus* infections using household decolonization strategies was only partially successful [23]. In this study both mupirocin and chlorhexidine were used to eradicate colonization from the nares as well as other body sites. Subjects in households where all members underwent decolonization still had a 28% and 38% rate of recurrent infections at 3 and 6 months, respectively. As noted by the authors, environmental contamination could help explain the failure of the intervention to prevent recurrent infection in the households [23]. Our finding provides additional evidence to support the hypothesis of Miller and Diep [28] suggesting that fomites constitute a risk for CA-*S. aureus* infections. Fomites potentially serve as reservoirs, constituting a source for re-colonization or infection of household members that, in turn, increases the risk of transmission to other household members.

We also found that environmental contamination with a colonizing or clinical infection strain was a risk factor for transmission separately among both case households and control households. This finding suggests that the environment is an important reservoir for household transmission of *S. aureus*, among a large diversity of strain types, and not exclusively among epidemic strains, such as USA300.

As documented in earlier studies, this community-based investigation demonstrated high levels of *S. aureus* transmission among case and control households [12], [15], [17], [29]. Although there were significant differences between case and control households for variables associated with index case eligibility, control households also reported high levels of skin or soft-tissue infection (63%), antibiotic use (61%), and hospitalization (26%) in the past 6 months. This is in addition to the high levels of environmental contamination. These results therefore demonstrate the high burden of *S. aureus* among households in general. This high burden of *S. aureus* and the high levels of antibiotic use, also observed previously in this community [17], [21], may potentially select for the high proportion of CA-MRSA infections being caused by epidemic strains, in this case USA300, among this community.

Among the numerous potential risk factors that we assessed other than environmental contamination (sociodemographic, health, hygiene, drug use and other household level risk factors), only the presence of a child under 5 was significantly and independently associated with transmission. The study design also allowed us to assess the role of infection on *S. aureus* transmission. Transmission among case households was increased when the clinical infection strain was included (*P<*.01). Recent antibiotic treatment presumably accounted for the low proportion of indexes that were colonized with the clinical infection strain. The lowered nasal colonization, in addition to the absence of cultures from other body sites, likely resulted in an underestimation of the true burden of *S. aureus* carriage [17], [29], [30]. Miller *et al.,* recently reported 50% of household members were colonized when multiple body sites were cultured [29]. However, looking exclusively at the spread of the clinical infection strain does not capture all of transmission. As we observed here, strains discordant from the clinical infection isolate were commonly found among case household members and transmission also occurred among control households, although at a significantly lower rate. Overall, the greater level of *S. aureus* colonization and transmission among case households suggests that CA-MRSA infection presents an additional *S. aureus* burden among households with an infection.

The epidemic strain USA300 was responsible for the excess burden of *S. aureus* in case households, accounting for the majority of CA-MRSA infections and environmental contaminants, a trend also noted in our earlier report [21]. Miller *et al.* recently found that infection of the household index with USA300 was an independent predictor of transmission [29]. These results suggest that USA300, in addition to its enhanced ability to cause infections, is an efficient colonizer of body sites as well as an environmental survivor. Taken together, the data suggest that household transmission of *S. aureus* is relatively common, regardless of the *S. aureus* strain. USA300, however, is unique in terms of its heightened invasiveness combined with its transmissibility.

There are a number of limitations to this study. These results are representative of a single predominantly Hispanic community in Northern Manhattan where the majority of index participants were female and may have limited generalizability. Second, this is a retrospective, observational study that uses a proxy variable as evidence of probable household transmission. Therefore, neither the directionality nor the source of transmission may be ascertained and the shared strains potentially indicate a shared exposure. Lastly, this study did not assess the impact of colonization of other body sites and, as noted above, colonization of other body sites is common [17], [29], [30]. Underestimating the colonization of *S. aureus*, and hence transmission, likely also underestimated the importance of environmental contamination as a reservoir. Although environmental contamination may provide a household reservoir and contribute to transmission, it may also serve as a surrogate marker of efficient colonization of multiple body sites [29], [31].

Notwithstanding these limitations, our findings suggest that environmental contamination plays a significant role in intra-household transmission. Environmental decontamination should be considered when treating CA-MRSA infections, particularly among households with multiple infected members. In light of the predominant role of the epidemic clone USA300, an additional strategy to prevent recurrent infections may be to target those epidemic strains that are both highly transmissible and more successful as pathogens that cause infections. This latter approach would allow a more efficient preventative strategy to be developed.
